# Hyaluronic acid fragments enhance the inflammatory and catabolic response in human intervertebral disc cells through modulation of toll-like receptor 2 signalling pathways

**DOI:** 10.1186/ar4274

**Published:** 2013-08-22

**Authors:** Lilian Quero, Marina Klawitter, Anja Schmaus, Melanie Rothley, Jonathan Sleeman, André N Tiaden, Juergen Klasen, Norbert Boos, Michael O Hottiger, Karin Wuertz, Peter J Richards

**Affiliations:** 1 Spine Research Group, CABMM, University of Zürich, Winterthurerstrasse 190, Zürich 8057, Switzerland; 2 AOSpine Research Network, Stettbachstrasse 6, Duebendorf 8600, Switzerland; 3 Bone and Stem Cell Research Group, CABMM, University of Zürich, Winterthurerstrasse 190, Zürich 8057, Switzerland; 4 Karlsruher Institut für Technologie (KIT), Hermann-von-Helmholtz-Platz 1, Eggenstein-Leopoldshafen 76344, Germany; 5 Universitätsmedizin Mannheim, University of Heidelberg, Centre for Biomedicine and Medical Technology Mannheim (CBTM), Theodor-Kutzer-Ufer 1-3, Mannheim 68167, Germany; 6 University Hospital Balgrist, Centre for Spinal Surgery, Forchstrasse 340, Zürich 8008, Switzerland; 7 Institute of Veterinary Biochemistry and Molecular Biology, University of Zürich, Winterthurerstrasse 190, Zürich 8057, Switzerland; 8 Zurich Centre for Integrative Human Physiology (ZIHP), University of Zürich, Winterthurerstrasse 190, Zürich 8057, Switzerland; 9 Institute for Biomechanics, Swiss Federal Institute of Technology (ETH), Wolfgang-Pauli-Strasse 10, Zürich 8093, Switzerland

## Abstract

**Introduction:**

Intervertebral disc (IVD) degeneration is characterized by extracellular matrix breakdown and is considered to be a primary cause of discogenic back pain. Although increases in pro-inflammatory cytokine levels within degenerating discs are associated with discogenic back pain, the mechanisms leading to their overproduction have not yet been elucidated. As fragmentation of matrix components occurs during IVD degeneration, we assessed the potential involvement of hyaluronic acid fragments (fHAs) in the induction of inflammatory and catabolic mediators.

**Methods:**

Human IVD cells isolated from patient biopsies were stimulated with fHAs (6 to 12 disaccharides) and their effect on cytokine and matrix degrading enzyme production was assessed using quantitative real-time polymerase chain reaction (qRT-PCR) and enzyme-linked immunosorbent assay (ELISA). The involvement of specific cell surface receptors and signal transduction pathways in mediating the effects of fHAs was tested using small interfering RNA (siRNA) approaches and kinase inhibition assays.

**Results:**

Treatment of IVD cells with fHAs significantly increased mRNA expression levels of interleukin (*IL*)-*1β*, *IL-6*, *IL-8*, cyclooxygenase (*COX*)-*2*, matrix metalloproteinase (*MMP*)*-1 *and *-13*. The stimulatory effects of fHAs on IL-6 protein production were significantly impaired when added to IVD cells in combination with either Toll-like receptor (*TLR*)*-2 *siRNA or a TLR2 neutralizing antibody. Furthermore, the ability of fHAs to enhance IL-6 and MMP-3 protein production was found to be dependent on the mitogen-activated protein (MAP) kinase signaling pathway.

**Conclusions:**

These findings suggest that fHAs may have the potential to mediate IVD degeneration and discogenic back pain through activation of the TLR2 signaling pathway in resident IVD cells.

## Introduction

Intervertebral disc (IVD) degeneration is considered to be a major contributory factor to the development of discogenic low back pain (LBP), a prevalent and costly musculoskeletal disorder [1,2]. Efforts to develop more effective therapies to combat this condition are hampered by the lack of information relating to the pathophysiological mechanisms responsible for instigating IVD degeneration and the ensuing LBP. There is, however, some evidence suggesting that elevated levels of various pro-inflammatory cytokines within degenerated IVDs may play a decisive role in mediating pain sensation [3-6]. Therefore, a better appreciation of the processes governing cytokine production within degenerated IVDs may help in the development of more effective treatment strategies to combat discogenic LBP.

Breakdown of the IVD extracellular matrix (ECM) is driven by a collection of proteolytic enzymes of which the matrix metalloproteinases (MMPs) and aggrecanases (members of the ADAMTS (A Disintegrin And Metalloproteinase with Thrombospondin Motifs) family) have been the most extensively studied [7-10]. These have the potential to degrade numerous matrix components as well as to give rise to a variety of reactive fragment species, which themselves may further act to stimulate and activate IVD cells. This is made evident by findings from our own studies, and from others, where proteolytic fragments of fibronectin and type II collagen have been shown to induce MMP expression in human IVD cells [11-14]. In addition to proteins and proteoglycans, numerous glycosaminoglycans (GAGs) also exist within the IVD, and include hyaluronic acid (HA), chondroitin sulfate and keratan sulfate, although only HA exists in the form of a free GAG [15]. Among these, HA has received significant attention due to the stimulatory nature of its degradation products on various cell types.

HA is a polymer composed of repeating disaccharide units comprised of D-glucuronic acid and D-N-acetylglucosamine. Whilst existing as a high molecular weight (HMW) polymer (>10^6 ^kDa) under normal conditions, HA can become degraded in response to various pathogenic events resulting in the generation of low molecular weight (LMW) fragments (fHAs) [16]. This may be brought about through the actions of various enzymes, such as hyaluronidases [17], as well as by exposure to non-enzymatic mediators, including reactive oxygen species (ROS) [18]. More specifically, pro-inflammatory agents, such as IL-1β, have been shown to induce the release and fragmentation of HA from cartilage explants [19]. This may be of particular relevance to the development of degenerative disc disease, where reductions in GAG content together with increases in IL-1β are wholly evident in degenerated IVDs [20,21]. Although there is currently no evidence confirming the presence of fHAs within disc tissue, it may be reasonable to assume that the sequence of catabolic and inflammatory events within the degenerating disc could provide an environment conducive to the production of fHAs. However, the potential involvement of such fragments in the pathogenesis of IVD degeneration has not yet been considered. Certainly, fHAs have the capacity to invoke both an inflammatory response as well as induce synthesis of tissue degrading enzymes when added to chondrocytes *in vitro *[22-25]. These effects are mediated through HA cell surface receptors CD44 and/or toll-like receptor (TLR)-4, with subsequent activation of NF-κB [24,25]. The receptor for hyaluronan-mediated motility (RHAMM, CD168) may also represent an additional means through which fHAs could mediate their stimulatory effects [26]. However, no studies have yet sought to investigate the influence of fHAs on the inflammatory and catabolic response in human IVD cells, and to assess their possible mode of action.

In the current report, we have set out to investigate the *in vitro *effects of fHAs on human IVD cells isolated from the discs of patients undergoing spine surgery. Small fHAs ranging in size from 12 to 24 mer were incubated with IVD cells and their influence on inflammatory and catabolic processes evaluated. Furthermore, studies were conducted in an attempt to identify the signalling pathways responsible for mediating the effects of fHAs. Our results clearly demonstrate that fHAs enhance both the pro-inflammatory and catabolic response in IVD cells, being mediated primarily through the TLR2 signaling pathway. These findings may be considered of significant clinical importance, based on the fact that increases in pro-inflammatory cytokine and MMP production are main features of IVD degeneration.

## Materials and methods

### Isolation and culture of IVD cells

Human IVD tissue was obtained from patients undergoing spinal surgery for symptomatic degenerative disc disease, disc herniation or spinal trauma following informed consent in accordance with the Ethics Committee of the Canton of Zurich (carried out at University Hospital Balgrist, Zurich, Switzerland) (Table 1) and IVD cells isolated and cultured as previously described [11]. Cells were used for experiments at passages 2 to 3.

**Table 1:** Details of patients used in the study.

Patient	Pathology	Severity Grade*^a^*	Disc Level
1	DH	4	L4/5
2	SD	5	L4/5
3	DH	5	L4/5
4	DH	5	C5/6
5	DH	5	L5/S1
6	DH	4	L4/5
7	DH	3	L5/S1
8	DH	5	L5/S1
9	DH	4	L4/5
10	DH	4	L4/5
11	DH	5	L4/5
12	DH	4	L4/5
13	DH	3	L2/3
14	DH	4	L5/S1
15	DH	4	L5/S1
16	DH	5	L5/S1
17	DH	3	L5/S1
18	DH	5	L4/5
19	DH	4	L5/S1
20	DH	4	C6/7
21	DH	4	L4/5
22	DH	5	L5/S1

*^a ^*The degree of IVD degeneration in patients was assessed prior to surgical intervention by magnetic resonance imaging (MRI) using a 5-level grading system based on Pfirrmann's classification of disc degeneration.

C, cervical; DH, disc herniation; F, female; L, lumber; M, male; S, sacral; SD, segment degeneration

### Preparation of HA fragments

Hyaluronic acid oligomers of 6 to 12 disaccharide units in length were prepared as previously described [27]. Ultrapure HMW HA (Healon 5), kindly provided by Amo (Ettlingen, Germany), was dissolved at 5 mg/ml in 0.3 M sodium phosphate buffer, pH 5.3, sonified and subsequently enzymatically digested with 200 U/ml bovine testis hyaluronidase (Sigma-Aldrich, Seelze, Germany) for six hours at 37°C. The resulting fragments were separated on a Bio Gel P10 column (3.5 × 115 cm) (BioRad, Munich, Germany) and 3 ml fractions collected. The concentration of HA in the fractions was determined by measuring the absorbance at 210 nm with reference to standards. The fractions were tested for endotoxin contamination using the Limulus Amebocyte Lysate (LAL)-assay kit (Lonza, Verviers, Belgium) according to the manufacturer's instructions. In all cases, endotoxin levels were below detection limits.

**Figure 1:**
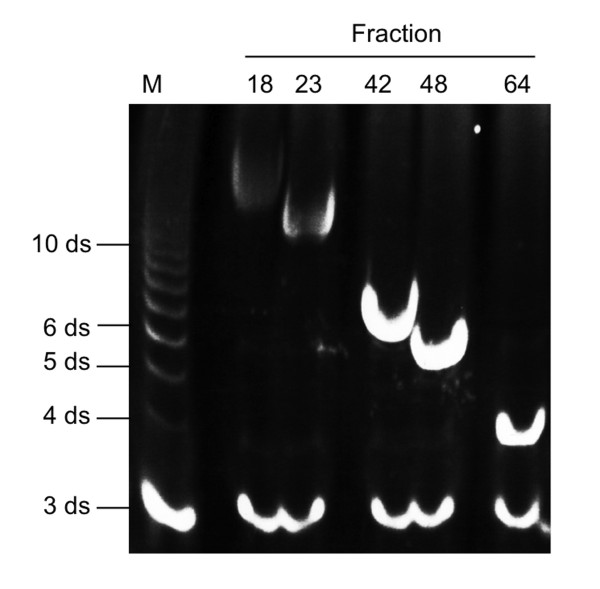
**Preparation of fHAs**. High molecular weight (HMW) hyaluronic acid (HA) was digested with bovine testis hyaluronidase. The resulting fragments were separated on a Bio Gel P10 column. For determination of fragment size fluorophore-assisted carbohydrate electrophoresis (FACE) analysis was performed. In this example, fractions 18, 23, 42, 48 and 64 derived from one HA-preparation were compared with a commercially-available HA standard (Sigma-Aldrich). M, HA standard marker; ds, disaccharide units.

For determination of HA fragment size, fluorophore-assisted carbohydrate electrophoresis (FACE) analysis of 7-amino-1,3-naphthalenedisulfonic acid (ANDS)-labeled fragments was performed. Briefly, samples were dried and resuspended in 5 µl 0.15 M ANDS (in 0.15% Acetic Acid) and 5 µl 1 M NaCNBH_4 _(in DMSO) (both Sigma-Aldrich). After 16 hours at 37°C the samples were dried and resuspended in 20% glycerine. Samples were separated on a 30% polyacrylamide gel at 15 mA. Bands were visualized in the gel by UV illumination. The size of the oligosaccharides in the fractions was determined by comparing the bands with similarly labeled, commercially available HA fragments of defined sizes (Sigma-Aldrich) (Figure 1).

### Gene expression profile in IVD cells treated with fHAs

IVD cells (1 × 10^6^) were cultured in 150 cm^2 ^flasks and starved in serum-free medium for 2 h prior to stimulation. Cells were incubated in medium alone or medium supplemented with fHAs (5 or 20 µg/ml) for up to 18 hours. Total RNA was isolated from IVD cells using the PureLink RNA Mini kit (Life Technologies, Zug, Switzerland) according to the manufacturer's recommendation (Life Technologies), then 1 μg total RNA was reverse-transcribed using Superscript II (Life Technologies). Quantification of mRNA expression was performed on the StepOnePlus Real-Time PCR System (Life Technologies) using the TaqMan Gene Expression Assays (Life Technologies) specific for *IL-1β*, *IL-6*, *IL-8*, *TNF-α*, *MMP-1*, *-2*, *-3*, *-9*, *-13*, *COX-2*, *ADAMTS4 *and *ADAMTS5 *(Table 2). Values were normalized to TATA-Box binding protein (*TBP*) mRNA levels and presented as either 2^-Δ*C*T ^or as fold change as compared to untreated cells according to the 2^-ΔΔ*C*T ^method where stated.

**Table 2:** TaqMan gene expression assays used for qRT-PCR.

Target gene	Assay ID
TATA box binding protein (*TBP*)	Hs00427620_m1
Interleukin 1 β (*IL-1β*)	Hs00174097_m1
Interleukin 6 (*IL-6*)	Hs00174131_m1
Interleukin 8 (*IL-8*)	Hs00174103_m1
Tumor Necrosis Factor α (*TNFα*)	Hs00174128_m1
Matrix metalloproteinase 1 (*MMP-1*)	Hs00233958_m1
Matrix metalloproteinase 2 (*MMP-2*)	Hs01548724_m1
Matrix metalloproteinase 3 (*MMP-3*)	Hs00968308_m1
Matrix metalloproteinase 9 (*MMP-9*)	Hs00957555_m1
Matrix metalloproteinase 13 (*MMP-13*)	Hs00233992_m1
Cyclooxygenase 2 (*COX-2*)	Hs00153133_m1
Aggrecanase 1 (*ADAMTS4, AD4*)	Hs00943031_g1
Aggrecanase 2 (*ADAMTS5, AD5*)	Hs00199841_m1

### Stimulation of IL-6 production in IVD cells

IVD cells (1.3 x10^5^) were cultured in 12-well plates and starved in serum-free medium for 2 h prior to stimulation. Cells were incubated in medium alone or medium supplemented with fHAs (20 µg/ml), Pam3CysSerLys4 (Pam3CSK4) (25 ng/ml) (LabForce, Nunningen, Switzerland), IL-1β (5 ng/ml) (Peprotech, London, UK) or lipopolysaccharide (LPS) (25 ng/ml) (**LuBioScience**, Luzern, Switzerland) for up to 18 hours. Culture supernatants were harvested for further analysis using a specific IL-6 ELISA (BD Biosciences, Allschwil, Switzerland).

### The effect of gene silencing on fHA-mediated IL-6 production in IVD cells

Specific knock down of *TLR2*, *TLR4*, *CD44 *and *RHAMM *expression was performed with small interfering (si)RNA oligos (Qiagen, Hombrechtikon, Switzerland). Human IVD cells (1.3 × 10^5 ^cells) were transfected with 10 or 20 nM of siRNA specific for *TLR2 *(SI00050036), *TLR4 *(SI04951149), *CD44 *(SI00299705), or *RHAMM *(SI04435347), or negative control siRNA (SI03650325) using lipofectamine RNAiMAX (Life Technologies) in 12-well plates. Following transfection, cells were incubated with fresh growth medium (without antibiotics) and incubated for 24 hours at 37°C, 5% CO_2_. Cells were then stimulated with fHAs (20 μg/ml) for 18 hours and culture supernatants harvested for further analysis using a specific IL-6 ELISA (BD Biosciences).

### The effect of TLR2 inhibition on fHA-mediated IL-6 production in IVD cells

IVD cells (1.3 × 10^5^) were cultured in 12-well plates and starved in serum-free medium for 2 h prior to stimulation. Cells were then pre-incubated for one hour with either an affinity purified polyclonal rat anti-human TLR2 neutralizing antibody (final concentration 5 µg/ml) (LabForce, Switzerland) or an isotype matched IgG control (Lucerna-Chem, Luzern, Switzerland). Cells were then stimulated with fHAs (20 μg/ml) or Pam3CSK4 (25 ng/ml) for 18 hours and culture supernatants harvested for further analysis using a specific IL-6 ELISA (BD Biosciences).

### The role of NF-κB in fHA-dependent IVD cell activation

IVD cells (3 × 10^5^) were cultured in six-well plates and starved in serum-free medium for 2 h prior to stimulation. Cells were then treated for up to one hour with either fHAs (20 μg/ml) or IL-1β (5 ng/ml). For the detection of NF-κB (p65) by immunofluorescence, cells were fixed with ice cold methanol (-20°C) for 10 minutes at selected time points, blocked for 10 minutes with PBS containing 1% BSA (Sigma) and 0.1% Triton-X100 (Sigma), and incubated with polyclonal rabbit anti-NF-κB (p65) (Santa Cruz Biotechnologies, Heidelberg, Germany) (1:200) for one hour at room temperature. NF-κB (p65) was detected using goat anti-rabbit Cy2 (Jackson ImmunoResearch, Newmarket, Suffolk, UK) (1:200) and visualized by fluorescence microscopy. For Western blot analysis, cells were first washed with buffer containing 10 mM HEPES (pH 7.9), 1.5 mM MgCl_2_, 10 mM KCl, 1 mM PMSF, 5 mM DTT with freshly added protease inhibitor cocktail (Sigma-Aldrich) and then lysed with 0.1% NP-40 for five minutes on ice. Nuclear pellets were harvested after centrifugation at 10,000 rpm for 5 minutes at 4°C, and lysed for 20 minutes in buffer containing 20 mM HEPES (pH 7.9), 1.5 mM MgCl_2_, 420 mM NaCl, 25% glycerol, 1 mM PMSF and 5 mM DTT. Protein concentrations were determined using the Bradford Assay (BioRad) and equal amounts loaded onto 12% SDS-PAGE gels. Protein was subsequently electroblotted onto PVDF membranes and incubated with nonfat dry milk (5%), 50 mM Tris-HCl, pH 7.6, 150 mM NaCl, 0.1% Tween 20 (TBST) for one hour at room temperature. Membranes were then incubated for 2 hours at room temperature with either anti- NF-κB (p65) (1:200) or anti-PARP1 (1:1,000) (both from LabForce, Switzerland). After washing in TBST three times for five minutes each, membranes were incubated with an appropriate HRP-conjugated secondary antibody for one hour at room temperature. Following a further washing step, peroxidase activity was detected using SuperSignal West Dura Chemiluminescent Substrate (Thermo Scientific, Lausanne, Switzerland). NF-κB (p65) binding activity was measured in nuclear extracts from IVD cells using the NF-κB (p65) Transcription Factor Assay according to the manufacturer's recommendations (Cayman, Tallinn, Estonia). All absorbance measurements were carried out at 655 nm.

### Role of MAP kinases in mediating the effects of fHAs in IVD cells

Cultured IVD cells were treated for 15 minutes with fHAs (20 μg/ml), IL-1β (5 ng/ml), LPS (25 ng/ml) or left untreated and whole protein cell extracts isolated following lysis in buffer containing 50 mM HEPES (pH 7.5), 450 mM NaCl, 15% glycerol, 2 mM EDTA, 1 mM PMSF and a freshly added protease inhibitor cocktail (Sigma-Aldrich). Protein was harvested following centrifugation at 14,000 rpm for 30 minutes and equal amounts loaded onto 12% SDS-PAGE gels and transferred to PVDF. Membranes were then incubated for two hours with either rabbit anti-p38 (1:1,000), rabbit anti-phospho-p38 (Thr180/Tyr182) (1:1,000), rabbit anti-p44/44 (1:1,000), rabbit anti-phospho-p44/42 (1:1,000), rabbit anti-SAPK/JNK (1:1,000) or rabbit anti-phospho-SAPK/JNK (Thr183/Tyr185) (1:200) (all from Cell Signaling Technology, Allschwil, Switzerland) and further analysed as described above. The functional role of MAP kinases in mediating the effects of fHAs was investigated using MAP kinase inhibitors. IVD cells were treated with fHA (20 μg/ml) alone (Control) or in combination with MAP kinase inhibitors (10 μM) specific for p38 (SB203580), ERK 1/2 (PD98059) or SAPK/JNK (SP600125) and culture supernatants harvested for further analysis using ELISAs specific for IL-6 (BD Biosciences) or MMP-3 (R and D Systems, Abingdon, UK) according to the manufacturer's protocol.

### Statistical analysis

All statistical analyses were carried out using SPSS19.0 (SPSS Inc., Chicago, IL, USA). Data were first assessed for normality of distribution using the Kolmogorov-Smirnov test. Parametric analysis of normally distributed data was performed using the two-tailed unpaired Student's *t*-test or one-way analysis of variance (ANOVA) followed by Tukey's *post-hoc *tests for multiple group comparisons. Non-parametric data were analyzed using the Kruskal-Wallis one-way analysis of variance for multiple group comparisons followed by the Mann-Whitney U test for comparisons between two groups. A *P*-value of <0.05 was considered statistically significant. All data were expressed as mean ± standard deviation (S.D.).

## Results

### Effect of fHAs on the expression of inflammatory and catabolic genes in IVD cells

**Figure 2:**
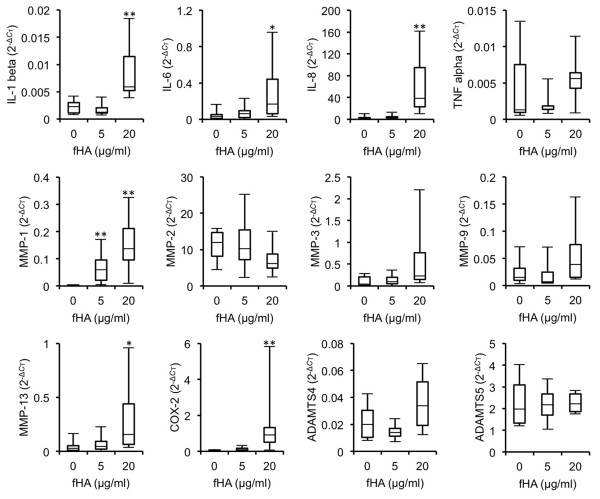
**Gene expression profile in IVD cells treated with fHAs**. Human IVD cells were incubated for 18 hours with hyaluronic acid fragments (fHAs) at either 5 or 20 μg/ml (*n *= 4 to 6) and RNA harvested for analysis by qRT-PCR. Values were normalized to TATA-Box binding protein (*TBP*) mRNA and expressed as 2^-Δ*C*T^. Statistical analysis was performed using the Kruskal-Wallis one-way analysis of variance for multiple group comparisons followed by the Mann-Whitney U test for comparisons between two groups, * *P *<0.05, ** *P *<0.01 as compared to untreated IVD cells. *IL*, interleukin; *TNF*, tumor necrosis factor; *MMP*, matrix metalloproteinase; *COX*, cyclooxygenase; *ADAMTS*, A Disintegrin And Metalloproteinase with Thrombospondin Motifs.

Initial experiments were undertaken in order to assess the effects of fHAs on the expression of a range of inflammatory and catabolic genes in cultured IVD cells. Treatment of IVD cells was carried out for up to 18 hours using concentrations of fHAs based on previous studies [24,28]. Stimulation of cells with fHAs at either 5 or 20 μg/ml resulted in significant alterations in several of the genes analysed (Figure 2). The most noticeable effects were observed in cells treated with the higher dose of fHAs (20 μg/ml), where significant increases in expression levels were measured for *IL-1β *(*P *<0.01), *IL-6 *(*P *<0.05), *IL-8 *(*P *<0.01), *MMP-1 *(*P *<0.01), *MMP-13 *(*P *<0.05) and *COX-2 *(*P *<0.01). All subsequent experiments therefore involved the use of fHAs at 20 μg/ml.

### The stimulatory effect of fHAs on IVD cells is dependent on functionally active TLR2

Of the genes identified as being regulated by fHAs, *IL-6 *was considered an appropriate candidate for further investigations based on its roles as both a pro-inflammatory cytokine and also as a mediator of pain [3].

**Figure 3:**
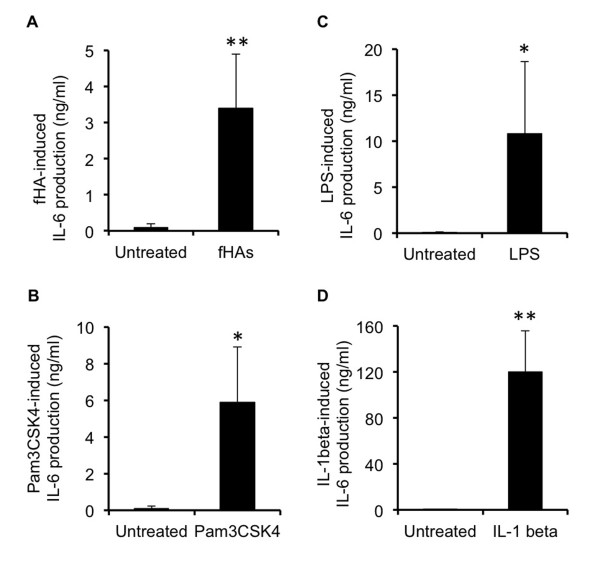
**Stimulation of IL-6 production in IVD cells**. Human intervertebral disc (IVD) cells were incubated for 18 hours with either: hyaluronic acid fragments (fHAs) (20 μg/ml) (*n *= 4) (**A**); Pam3CSK4 (25 ng/ml) (*n *= 4) (**B**); lipopolysaccharide (LPS) (25 ng/ml) (*n *= 5) (**C**); or interleukin (IL)-1β (5 ng/ml) (*n *= 5) (**D**), and IL-6 protein levels determined in supernatants using a specific ELISA. In all cases, analyses were performed in triplicate and values expressed as mean ± S.D. Statistical analysis was performed using the Student's *t*-test, * *P *<0.05, ** *P *<0.01 as compared to untreated IVD cells.

Stimulation of IVD cells with fHAs induced a significant increase in IL-6 protein production (3.5 ± 1.5 ng/ml; *P *<0.01) as compared to untreated cells (Figure 3A), as did other well-known instigators of IL-6 production, including toll-like receptor activators Pam3CSK4 (6.0 ± 3 ng/ml) (Figure 3B) and LPS (10.8 ± 7.85 ng/ml) (Figure 3C), as well as the pro-inflammatory cytokine IL-1β (120 ± 36 ng/ml) (Figure 3D).

**Figure 4:**
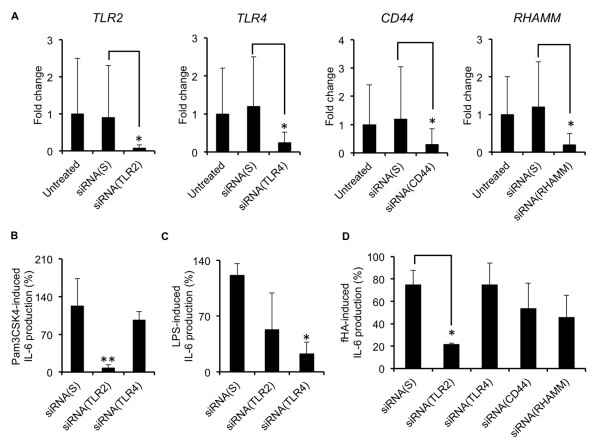
**The effect of gene silencing on fHA-mediated IL-6 production in IVD cells**. **A**) siRNA-mediated knockdown of genes Toll like receptor (*TLR*)*2*, *TLR4*, *CD44 *and *RHAMM *was confirmed after 30 hours in intervertebral disc (IVD) cells by qRT-PCR (*n *= 4). In each case, gene expression was calculated as fold change as compared to untreated cells. The use of a non-specific scrambled siRNA (siRNA (S)), confirmed specificity of gene knockdown. **B, C**) Interleukin (IL)-6 protein production by Pam3CSK4- (25 ng/ml) (*n *= 4) (**B**) and lipopolysaccharide (LPS)- (25 ng/ml) (*n *= 3). (**C**) stimulated IVD cells following gene knockdown of *TLR2 *or *TLR4 *respectively, as determined by IL-6 ELISA. (**D**) hyaluronic acid fragment (fHA)-treated (20 μg/ml) IVD cells following gene knockdown as determined by IL-6 ELISA (*n *= 4). IL-6 protein levels are represented as a percentage of those measured for untreated cells. In all cases, analyses were performed in triplicate and values expressed as mean ± S.D. Statistical analysis was performed using the Student's *t*-test, * *P *<0.01.

An siRNA approach was then used to target genes encoding the cell surface receptors *TLR2*, *TLR4*, *CD44 *and *RHAMM*, with the aim of identifying potential receptors involved in engaging fHAs. Knockdown efficiency was confirmed in IVD cells after 24 hours using qRT-PCR (Figure 4A). TLR2 and TLR4 were selected for further evaluation of functional loss of receptor activity. Confirmation of efficient and comparable TLR2 and TLR4 loss-of-function was substantiated in siRNA-treated cells through examination of their ability to express IL-6 following incubation with TLR ligands Pam3CSK4 (Figure 4B) and LPS (Figure 4C). fHA-dependent IL-6 production by IVD cells was also significantly reduced following TLR2 loss-of-function (Figure 4D), although no significant reductions in IL-6 production were observed in cells in which TLR4, CD44 or RHAMM had been knocked down.

**Figure 5:**
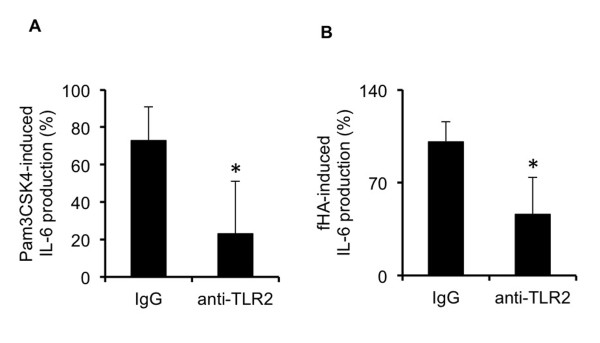
**The effect of TLR2 inhibition on fHA-mediated IL-6 production in IVD cells**. Interleukin (IL)-6 protein production by Pam3CSK4- (25 ng/ml) (**A**) and hyaluronic acid fragment (fHA)-treated (20 μg/ml) (**B**) intervertebral disc (IVD) cells following antibody-mediated neutralization of Toll like receptor (TLR)2 activity (*n *= 4). IL-6 protein levels are represented as a percentage of those measured for untreated cells. In all cases, analyses were performed in triplicate and values expressed as mean ± S.D. Statistical analysis was performed using the Student's *t*-test, * *P *<0.05 as compared to cells treated with non-specific IgG control antibody.

Further confirmation of TLR2's involvement in the activation of IVD cells by fHAs was demonstrated in antibody-mediated neutralization studies. Initial studies confirmed the effectiveness of the polyclonal anti-TLR2 antibody to neutralize TLR2 activity as demonstrated by its ability to suppress Pam3CSK4-dependent IL-6 production as compared to a non-specific IgG control antibody (Figure 5A). Similarly, antibody-mediated TLR2 inactivation also significantly reduced (*P *<0.05) the stimulatory effects of fHAs on IL-6 production by IVD cells (Figure 5B).

### The role of NF-κB in fHA-mediated IVD activation

Activation of NF-κB is considered to be a primary means through which fHAs mediate their stimulatory effects in chondrocytes [24,25]. We, therefore, carried out a series of experiments to investigate whether fHAs could also induce NF-κB activation in human IVD cells.

**Figure 6:**
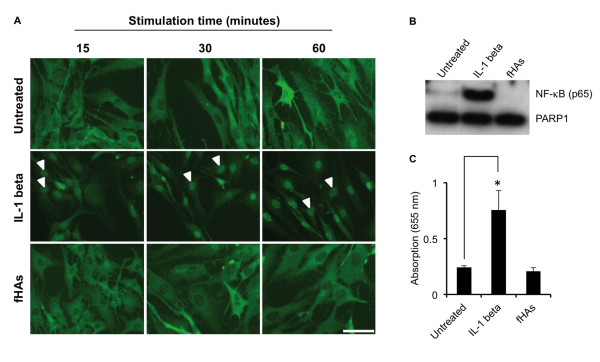
**The role of NF-κB in fHA-dependent IVD cell activation**. (**A**) Immunofluorescence staining of intervertebral disc (IVD) cells for NF-κB (p65) (green) following treatment with either hyaluronic acid fragments (fHAs) (20 μg/ml) or interleukin (IL)-1β (5 ng/ml) at selected time points. Nuclear NF-κB (p65) is indicated by arrowheads. Untreated IVD cells served as a control. Scale bar = 50 μm. (**B**) Western blot analysis of nuclear extracts from untreated IVD cells or cells treated for 1 hour with either fHAs (20 μg/ml) or IL-1β (5 ng/ml). Levels of PARP1 protein served as a loading control. (**C**) NF-κB (p65) DNA binding activity in nuclear extracts from untreated IVD cells, or cells treated for one hour with IL-1β or fHAs (20 μg/ml) (*n *= 3). In all cases, analyses were performed in triplicate and values expressed as mean ± S.D. Statistical analysis was performed using the Student's *t*-test, * *P *<0.01 as compared to untreated cells.

We were unable to observe any evidence of NF-κB activation in IVD cells following stimulation with fHAs (20 μg/ml). This was clearly demonstrated by the lack of any increase in nuclear p65 as determined by both immunofluorescence staining (Figure 6A) and also Western blot analysis (Figure 6B) as compared to untreated cells. Furthermore, nuclear extracts harvested from IVD cells treated with fHAs demonstrated no significant increase in NF-κB (p65) DNA binding activity when tested using a specific transcription factor assay (Figure 6C). This was in direct contrast to cells treated with IL-1β, where obvious increases in nuclear p65 were evident in all of the performed analyses.

### Activation of IVD cells by fHAs is dependent on MAP kinase signalling pathways

**Figure 7:**
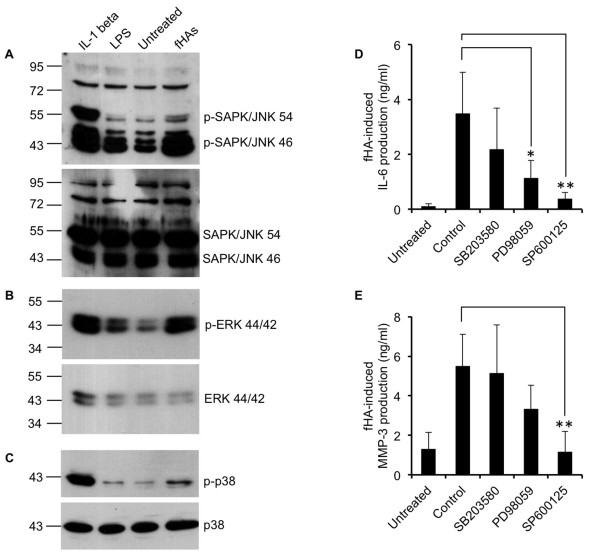
**Role of MAP kinases in mediating the effects of fHAs in IVD cells**. Cultured intervertebral disc (IVD) cells were treated for 15 minutes with hyaluronic acid fragments (fHAs) (20 μg/ml), interleukin (IL)-1β (5 ng/ml), lipopolysaccharide (LPS) (25 ng/ml) or left untreated and whole cell extracts harvested for Western blot analysis using antibodies specific to phosphorylated and non-phosphorylated SAPK/JNK (54/46) (**A**), ERK 1/2 (44/42) (**B**) and p38 (**C**). (**D, E**) IVD cells were treated with fHA (20 μg/ml) alone (Control) or in combination with MAP kinase inhibitors (10 μM) specific for p38 (SB203580), ERK 1/2 (PD98059) or SAPK/JNK (SP600125) and IL-6 (D) and matrix metalloproteinase (MMP)-3 (E) protein levels determined by specific ELISA (*n *= 4). In all cases, analyses were carried out in triplicate and values expressed as mean ± S.D. Statistical analysis was performed using Students *t*-test, * *P *<0.05, ** *P *<0.01 as compared to cells treated with fHAs alone (Control).

Based on the fact that TLR2 signalling also partly relies on the MAP kinase pathway [29], we next investigated whether fHAs had the capacity to activate various MAP kinases in IVD cells. Western blot analysis revealed a marked increase in the level of phosphorylated c-Jun-N-terminal kinase/stress-activated protein kinase (JNK/SAPK) (Figure 7A). In addition, we also saw a noticeable increase in the level of phosphorylated extracellular signal-regulated kinase (ERK) 1/2 (p44/p42) in cells treated with fHAs (Figure 7B). However, by comparison, only marginal increases in the level of p38 MAP kinase phosphorylation were observed in response to fHAs (Figure 7C).

In order to further clarify the potential involvement of MAP kinase activity in regulating fHA-dependent IVD cell activation, we performed additional experiments utilizing specific MAP kinase inhibitors. The stimulatory effects of fHAs on IL-6 production by IVD cells were significantly reduced following pre-exposure of the cells to either ERK inhibitor PD98059 (*P *<0.05) or JNK inhibitor SP600125 (*P *<0.01) (Figure 7D). The p38 MAP kinase inhibitor, SB203580, had only a weak inhibitory effect on fHA-dependent IL-6 production, which did not reach statistical significance (*P *= 0.27). We also investigated the ability of MAP kinase inhibitors to affect fHA-induced MMP production in IVD cells. As with IL-6, MMP-3 protein production was up-regulated by over four-fold (*P *<0.01) in IVD cells treated with fHAs (Figure 7E) and was significantly reduced following inhibition of JNK activity (*P *<0.01).

## Discussion

The primary source of discogenic back pain has been debated over the years, although there is now a growing body of evidence suggesting a close causal relationship between pro-inflammatory cytokine expression and the development of pain within the degenerated IVD [3,10,30]. Certainly, pro-inflammatory mediators have the capacity to provoke pain sensation [4-6], although it remains unclear as to which processes are responsible for initiating the ensuing cascade of cytokines in diseased discs. Considering the fact that discogenic back pain occurs primarily in degenerated discs, it is highly likely that the processes governing disc degeneration also play a role in instigating pro-inflammatory cytokine production.

Degradation of the ECM through catabolic processes can result in the generation of a variety of matrix protein fragments with the potential to influence cellular behavior in numerous tissue types, usually with a detrimental outcome [9,11,12,31-33]. Furthermore, fragments generated from HA have the potential to induce a number of pro-inflammatory responses as evidenced by their ability to up-regulate chemokines, cytokines and matrix degrading enzymes in several different cell types, including chondrocytes [22-27,34-36]. In the present report, we have extended these observations to include human IVD cells, where fHA-mediated stimulation was shown to significantly enhance the expression of pro-inflammatory cytokines *IL-1β*, *IL-6 *and *IL-8*. Moreover, fHAs were also found to stimulate the expression of certain matrix degrading enzymes, including *MMP-1*, *MMP-3 *and *MMP-13*, although significance was not always attained due to the large variations between patient samples. It is envisaged that larger population studies may help to resolve this issue and thus allow for a more accurate assessment of potential fHA-target genes. The induction of such matrix degrading enzymes by fHAs would undoubtedly contribute to IVD catabolism and thus perpetuate the on-going destructive processes within the actively degenerating disc. In addition to increases in mRNA expression levels, we were also able to detect enhanced levels of secreted IL-6 protein following fHA stimulation of IVD cells. This is considered to be of particular importance when assessing the relevance of fHAs in IVD degeneration *in vivo*, where secreted cytokines are the main protagonists in driving the pain sensation process [3,37].

The pro-inflammatory effects of LMW fHAs on chondrocytes are generally thought to be dependent on their interaction with TLR4 and CD44 [24,25]. In the present report, we utilized both gene silencing and antibody-directed inhibition approaches in an attempt to identify potential signaling pathways responsible for fHA-dependent IVD cell activation. Contrary to expectations, functional loss of either TLR4 or CD44 did not significantly influence fHA-induced IL-6 production in IVD cells. Furthermore, loss-of-function studies involving RHAMM, another potential mediator of fHA-dependent signaling, were also unable to demonstrate any significant decreases in IL-6 secretion by fHA-stimulated IVD cells. However, the stimulatory effects of fHAs were significantly decreased in IVD cells in which TLR2 expression had been effectively suppressed following siRNA treatment. These observations were further corroborated by studies in which TLR2 activity was inhibited through the use of a specific neutralizing antibody. To our knowledge, this is the first report confirming the involvement of TLR2 in fHA-induced cytokine production in human IVD cells.

The NF-κB signal transduction pathway has previously been implicated as a primary means through which fHAs mediate their effects [34,38]. More specifically, NF-κB activation has been reported to mediate the stimulatory effects of fHAs in chondrocytes [22,25]. In addition to NF-κB, signaling pathways involving MAP kinases have also been shown to play a functional role in the transduction of fHA signals [22,34]. In the present report, we were unable to demonstrate NF-κB activation in human IVD cells following treatment with fHAs, although convincing data were obtained which strongly implicated the MAP kinase pathway as being an important regulator of fHA signaling. We could demonstrate strong activation of MAP kinases ERK and JNK in IVD cells following short-term stimulation with fHAs, although p38 appeared to be less responsive to the actions of fHAs. These findings may have important ramifications in terms of identifying possible mechanisms through which fHAs induce both inflammatory and catabolic responses in IVD cells. Certainly, many of the genes up-regulated by fHAs in the current study have previously been confirmed as MAP kinase target genes in IVD cells [39-41]. Indeed, we were able to confirm activation of ERK and JNK but not p38 MAP kinases as being necessary requirements for fHA-mediated IL-6 production in IVD cells. Furthermore, increases in MMP-3 protein production due to fHAs also appeared to be dependent on JNK MAP kinase activity. This may have significant implications when considering therapeutic strategies for treating IVD degeneration and inflammatory pain development, and may, therefore, warrant further investigations into the possible clinical benefits of using MAP kinase inhibition for treating this debilitating disease.

## Conclusions

In conclusion, the data provided in the current report provide convincing evidence that fHA-dependent stimulation of human IVD cells is primarily regulated through TLR2-mediated activation of the MAP kinase pathway. Furthermore, our findings offer new insights into the potential molecular mechanisms governing IVD inflammatory pain development in patients with IVD degeneration. Clearly, therefore, further studies are now needed in order to confirm the presence and concentration of fHAs in tissue samples, and thereby allow for an accurate assessment of their true physiological role in IVD degeneration.

## Abbreviations

ADAMTS: A Disintegrin And Metalloproteinase with Thrombospondin Motifs; AND: 7-amino-1,3-naphthalenedisulfonic acid; COX: cyclooxygenase; ECM: extracellular matrix; ERK: extracellular signal-regulated kinase; fHAs: hyaluronic acid fragments; HA: hyaluronic acid; IL: interleukin; IVD: intervertebral disc; JNK/SAPK: c-Jun-N-terminal kinase/stress-activated protein kinase; LBP: low back pain; LMW: low molecular weight; LPS: lipopolysaccharide; MAP: mitogen-activate protein; MMP: matrix metalloproteinase; NF-κB: nuclear factor kappa B; RHAMM: receptor for hyaluronan-mediated motility; TBP: TATA-Box binding protein; TLR: toll-like receptor; TNF: tumour necrosis factor.

## Competing interests

The authors declare that they have no competing interests.

## Authors' contributions

LQ participated in all experimental studies and was involved in drafting the manuscript. MK participated in cell isolation and Western blot analyses. AS, MR and JS generated, purified and characterized the HA fragments used in the current study. ANT participated in all siRNA studies. JK participated in the acquisition of IVD cells used in the current study. NB and KW made substantial contributions to the conception and design of the study, and acquisition of funding. MOH made substantial contributions to the conception and design of the study, and interpretation of data. PJR made substantial contributions to the conception and design of the study, interpretation of data, and drafting of the manuscript. All authors read and approved the final manuscript version.
